# Fruit quality prediction based on soil mineral element content in peach orchard

**DOI:** 10.1002/fsn3.2794

**Published:** 2022-02-28

**Authors:** Hailong Sun, Xiao Huang, Tao Chen, Pengyu Zhou, Xuexi Huang, Weixin Jin, Dan Liu, Hongtu Zhang, Jianguo Zhou, Zhongjun Wang, Faisal Hayat, Zhihong Gao

**Affiliations:** ^1^ Research Institute of Pomology Chinese Academy of Agricultural Sciences Key Laboratory of Biology and Genetic Improvement of Horticultural Crops Germplasm Resources Utilization Ministry of Agriculture Xingcheng China; ^2^ 70578 College of Horticulture Nanjing Agricultural University Nanjing China; ^3^ Agricultural Service Center of Yangshan Town Wuxi China; ^4^ Xinyi Agriculture and Rural Affairs Bureau Xinyi China; ^5^ Agricultural Technology Extension Center of Wujin District Changzhou China; ^6^ Yancheng Biological Engineering Higher Vocational School Yancheng China

**Keywords:** artificial neural network, fruit quality, mineral element nutrition, peach, soil

## Abstract

Mineral nutrition of orchard soil is critical for the growth of fruit trees and improvement of fruit quality. In the present study, the effects of soil mineral nutrients on peach fruit quality were studied by using artificial neural network model. The results showed that the four established ANN models had the highest prediction accuracy (*R* ^2^ = .9735, .9607, .9036, and .9440, respectively). The results of prediction model sensitivity analysis showed that available B, Ca, N, and K in the soil had the greatest influence on the single fruit weight, available Fe, K, B, and Ca in the soil had the greatest effect on fruit soluble solid content, available Ca, N, B, and K in the soil had the greatest influence on the fruit titratable acid content, and available Ca, Fe, N, and Mn in the soil had the greatest effect on fruit edible rate. The response surface methodology analysis determined the optimal range of these mineral elements, which is critical for guiding precision fertilization in peach orchards and improving peach fruit quality.

## Graphical abstract

Through the determination of soil mineral nutrients and peach fruit quality, the artificial neural network models were established, and the model sensitivity analysis was conducted to obtain significant mineral elements, which further improved the suitable range for surface analysis to explore mineral elements.

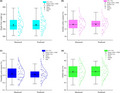

## 1 INTRODUCTION

Peach (*Prunus Persica* (L.) Batsch) was one of the most valuable stone fruit crops in the Rosaceae family (Wang et al., 2022), native to the northwest of China. It is currently the third‐largest deciduous fruit tree in China (Yu et al., 2019), second only to apples and pears. It has been cultivated in over 80 countries worldwide for its economic, social, and ecological benefits (Tian, 2020). Peach is a fruit with high nutritional value, rich in mineral elements, protein, sugar, fat, vitamins, and other nutrients, and is deeply loved by people (Serra et al., 2020; Wang, et al., 2021; Wang, Liu, et al., 2021; Yu et al., 2010).

Fruit quality was the central goal of fruit cultivation technology. Fruit's competitiveness in the market can only be improved by good fruit quality. In recent years, with the continuous improvement of people's living standards, they also raised their expectation for the nutritional value and fruit quality (Zhao et al., 2018). Several studies found that the soil mineral element content in orchard had a significant impact on fruit quality. The multivariate analysis of soil nutrients and fruit quality of kiwi orchard revealed that the soluble sugar of kiwi fruit was mainly affected by the available potassium and available sulfur, and the titratable acid was mainly affected by the organic matter (Chen et al., 2021). The nitrogen content of orchard soil directly affects the fruit quality and yield of peach (Zhu et al., 2019), there was a certain correlation between soil organic content, available potassium content, and peach fruit weight (Wang, Zhao, et al., 2021; Wang, Liu, et al., 2021). The correlation analysis between soil mineral nutrients and fruit quality of Jinsha pomelo showed a negative correlation between total sugar and available iron in the soil, and a negative correlation between edible rate and available manganese in soil (Yu et al., 2021). Currently, the research on fruit quality and mineral nutrients at home and abroad was only a simple difference and correlation analysis, which are incapable of revealing its complex internal relationship.

Artificial neural network (ANN) was a mathematical model, based on the basic principle of neural network in biology. After comprehending the structure of human brain and the response mechanism of external stimuli, it is possible to simulate the processing mechanism of complex information of the human brain nervous system using knowledge of network topology (Saffari et al., 2009). A complex network system was formed by many simple processing units (called neurons) connected with each other. As the basis of deep learning, neural network model played an important role. The neural network was an extensive parallel network composed of adaptive simple units, which can simulate the interaction between biological neural system and real‐world objects. It mainly consisted of three layers: input, hidden, and output (Tracey et al., 2011). It can make the machine recognize the pattern and trend of data through a special algorithm, and successfully predict and classify. Because of its good fault tolerance and good self‐learning ability, it had attracted the attention of scholars in many fields. In recent years, the ANN was more and more widely applied in the field of agriculture. Response surface methodology and artificial neural network could optimize the extraction of polysaccharides and polyphenols from blackcurrant fruit (Bu et al., 2021). The ANN model was used to predict and optimize the main quality parameters of corn for ethanol production (Voca et al., 2021). The ANN model has been shown to be an effective and reliable forecasting tool in many studies (Azarmdel et al., 2020; Banga et al., 2020; Huang et al., 2021; Kumar et al., 2020).

The present study used various ANN models to study the effects of soil mineral nutrient content on peach fruit quality, and the suitable range of main mineral elements was identified, providing a theoretical basis for precise fertilization of peach orchards.

## 2 MATERIALS AND METHODS

### 2.1 Materials

The experiment was carried out in Wuxi, Yancheng, and Changzhou in Jiangsu Province, which were also the main peach planting areas of Jiangsu. We chose 75 peach orchards with basically the same cultivation and management level. The fertilization method was mainly to apply organic fertilizer in autumn, about 1.5–1.8 tons per mu (666.67 m^2^). Nitrogen, phosphorus, and potassium fertilizer were applied before the fruit expansion stage, about 15 kg, 8 kg, and 20 kg, respectively. The main cultivar of peach in orchard was "Hujingmilu", six healthy adults with the same growth status and medium crown size were randomly selected as sample plants in each orchard. Twenty to thirty fresh fruits with normal maturity and similar size were randomly collected from each orchard. The sampling orientation and canopy were all the same. Four points were determined in the east, west, south, and north directions under the crown drip line of each sampling tree, and the surface soil of 0–30 cm was drilled with a soil sampler. After removing the sundries, the obtained soil was mixed evenly. A sample of approximately 1 kg of soil was quartered, dried naturally in the laboratory, ground into powder, and stored in a marked sealed bag after passing through a 100‐mesh nylon sieve (Safa et al., 2018).

### 2.2 Experimental methods

The content of soil available N was extracted by the ion exchange resin bag method (Liu et al.,  2017), and then determined by AA3 continuous flow analyzer (Wang, 2020). The contents of available P, K, Ca, Mg, Fe, Mn, Cu, Zn, and B in soil were extracted by AB‐DTPA extraction method (Hao et al., 2016) and determined by Agilent 710 ICP‐OES inductively coupled plasma atomic emission spectrometry (Li et al., 2018; Huang et al., 2018).

We used the 1/10,000 electronic analytical balance to determine the single fruit weight of peach, the pal‐1 portable digital display sugar meter to determine the soluble solid content of peach, and the titratable acid content of peach was quantitatively measured via acid–base titration (Cao et al., 2007). The edible rate = (single fruit weight‐single fruit stone weight)/single fruit weight *100.

### 2.3 Statistical analysis and neural models building

The artificial neural network model was constructed using soil mineral element content as input layer and peach fruit quality index as output layer. During the model development process, we randomly use 70% of the data for model training, 15% of the data for model validation, and the remaining 15% of the data for model testing. Meanwhile, we preprocessed the original data using the following formula (Shabani et al., 2017):

(1) $$M_{n}=\frac{M‐M_{\min}}{M_{\max}‐M_{\min}}$$

**FIGURE 1:**
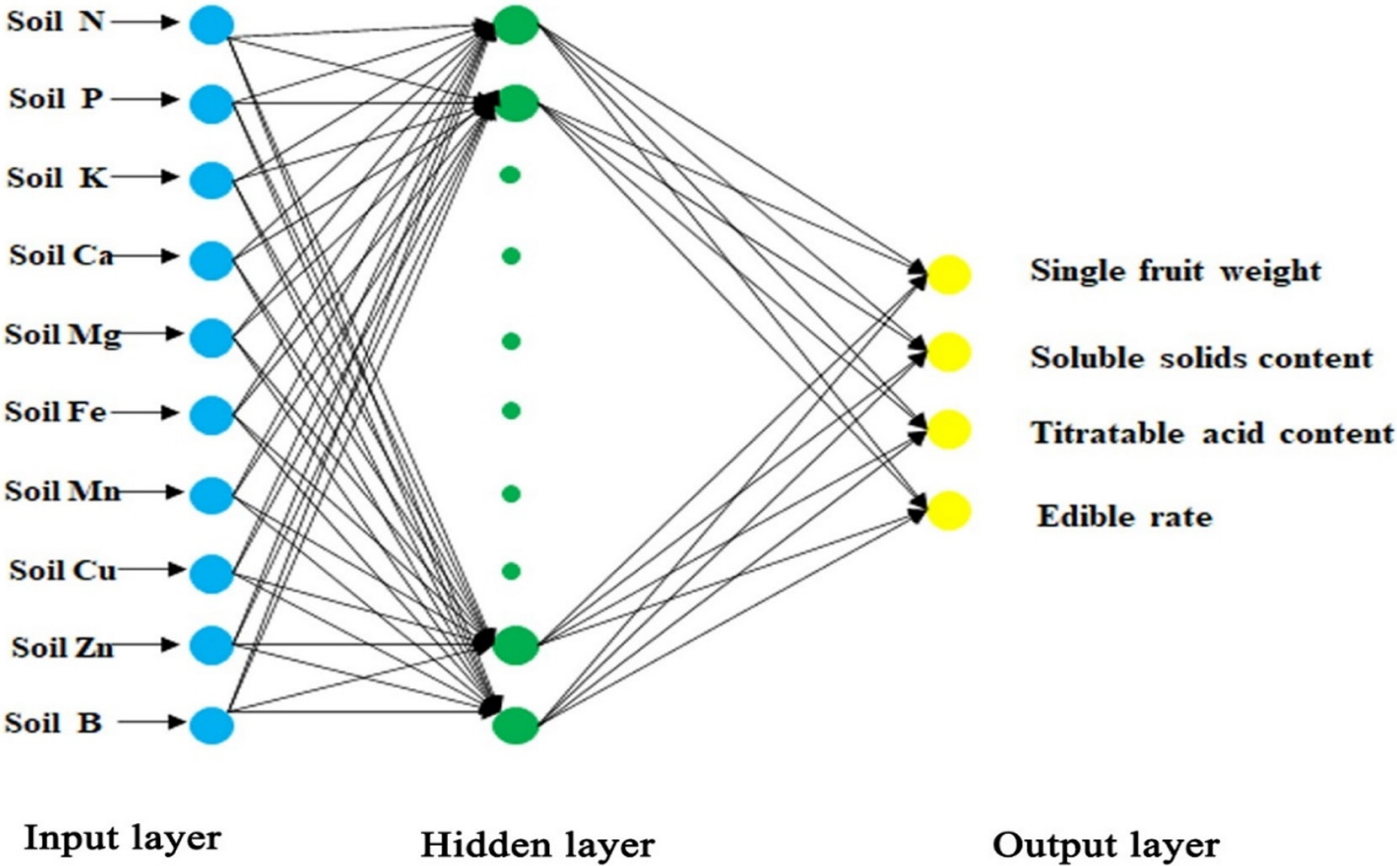
Three‐layer structure of artificial neural network (ANN) model

Where M is the original measurement value, M*_n_* is the preprocessed value, M*_max_* and M*_min_* are the maximum and minimum values of data. In the development process of the ANN system, we use three main structures (Figure 1). Meanwhile, we applied three different transfer functions and five different training functions (Tables 3, 4, 5) to establish the model through MATLAB software (version 2015) to find the final accurate prediction model. We also evaluated the performance of ANN model by some indexes, such as coefficient of determination (*R* ^2^), mean absolute error (MAE), relative standard error (RSE), mean square error (*MSE*), root mean square error (RMSE), and mean absolute percentage error (MAPE).

•Log‐sigmoid function:

(2) $$F\left(A\right)=\frac{1}{1+e^{A}}$$

•Tangent‐sigmoid function:

(3) $$F\left(A\right)=\frac{2}{1+e^{‐2A}}‐1$$

Linear function:

(4) $$F\left(A\right)=A$$

(5) $$R^{2}=\frac{\sum_{i=1}^{n}\left(Ti‐\bar{T}\right)\left(ti‐\bar{t}\right)}{\sqrt{\sum_{i=1}^{n}{\left(Ti‐\bar{T}\right)}^{2}\sum_{i=1}^{n}{\left(ti‐\bar{t}\right)}^{2}}}$$

(6) $$\text{RMSE}=\sqrt{\frac{1}{n}\sum_{i=1}^{n}{\left(Ti‐ti\right)}^{2}}$$

(7) $$\text{RSE}=\frac{\sqrt{\frac{1}{n}\sum_{i=1}^{n}{\left(Ti‐ti\right)}^{2}}}{\bar{T}}$$

(8) $$\text{MAE}=\frac{1}{n}\sum_{i=1}^{n}\left|Ti‐ti\right|$$

(9) $$\text{MAPE}=\frac{100\%}{n}\sum_{i=1}^{n}\left|\frac{Ti‐ti}{\mathrm{Ti}}\right|$$

Where *n* is the number of data, T*_i_* is the original measured values, t*_i_* is the predicted values of the established model, and the bar is the average value of the concerned variable. We constructed the best prediction model using neural network, and then eliminated model independent variables one by one to perform model sensitivity analysis. This allowed us to investigate the mineral elements that have a significant influence on fruit quality indexes. Finally, response surface analysis is used to determine the appropriate range of these mineral elements for the best fruit quality by analyzing the content of major mineral elements and the corresponding fruit quality indexes.

## 3 RESULTS

### 3.1 Fruit quality and soil mineral element content of peach orchards

The peach quality indicators of different orchards are shown in Table 1, the maximum value of the single fruit weight was 393.30 g, the minimum was 162.20 g, and the average value was 281.99 g. The maximum soluble solid content was 17.96%, the minimum was 9.60%, and the average value was 13.27%. The maximum value of the titratable acid content was 0.51%, the minimum was 0.14%, and the average value was 0.32%. The maximum value of the edible rate was 96.25%, the minimum was 92.70%, with an average value of 94.53%. Among them, the variation coefficient of titratable acid content was the largest (26.14%), and that of edible rate was the smallest (0.96%), indicating that the difference of titratable acid content in different orchards was significant; however, the difference of edible rate was minor.

**TABLE 1:** The fruit quality indicators of peach

	Single fruit weight (g)	Soluble solid content (%)	Titratable acid content (/%)	Edible rate (%)
Max	393.30	17.96	0.51	96.25
Min	162.20	9.60	0.14	92.70
Mean	281.99	13.27	0.32	94.53
STD	46.00	1.50	0.08	0.91
CV	16.31	11.28	26.14	0.96

The contents of soil mineral elements in different orchards are shown in Table 2. The available macro‐element average values of N, P, K, Ca, and Mg were 197.48 mg/kg, 71.33 mg/kg, 440.80 mg/kg, 220.96 mg/kg, and 154.20 mg/kg, respectively. The coefficient of variation of P and Mg was the largest, which indicated that there were great differences between the two macro‐elements in different orchards. The micro‐element average values of available Fe, Mn, Cu, Zn, and B were 168.07 mg/kg, 79.57 mg/kg, 9.15 mg/kg, 7.67 mg/kg, and 0.64 mg/kg, respectively. The coefficient of variation of Cu and Zn was the largest, which indicated that there were significant differences between the two micro‐elements in different orchards.

**TABLE 2:** The mineral element content of peach orchard soil

	N (mg/kg)	P (mg/kg)	K (mg/kg)	Ca (mg/kg)	Mg (mg/kg)	Fe (mg/kg)	Mn (mg/kg)	Cu (mg/kg)	Zn (mg/kg)	B (mg/kg)
Max	357.29	173.43	1250.84	310.34	493.00	279.09	234.81	58.83	39.68	1.13
Min	55.40	5.18	64.40	113.73	26.15	53.53	6.72	1.14	0.30	0.12
Mean	197.48	71.33	440.80	220.96	154.20	168.07	79.57	9.15	7.67	0.64
STD	56.99	41.75	213.40	43.97	89.47	58.70	35.85	8.73	7.57	0.26
CV	28.86	58.53	48.41	19.90	58.02	34.92	45.05	95.39	98.60	40.37

### 3.2 ANNs model for predicting the single fruit weight

**FIGURE 2:**
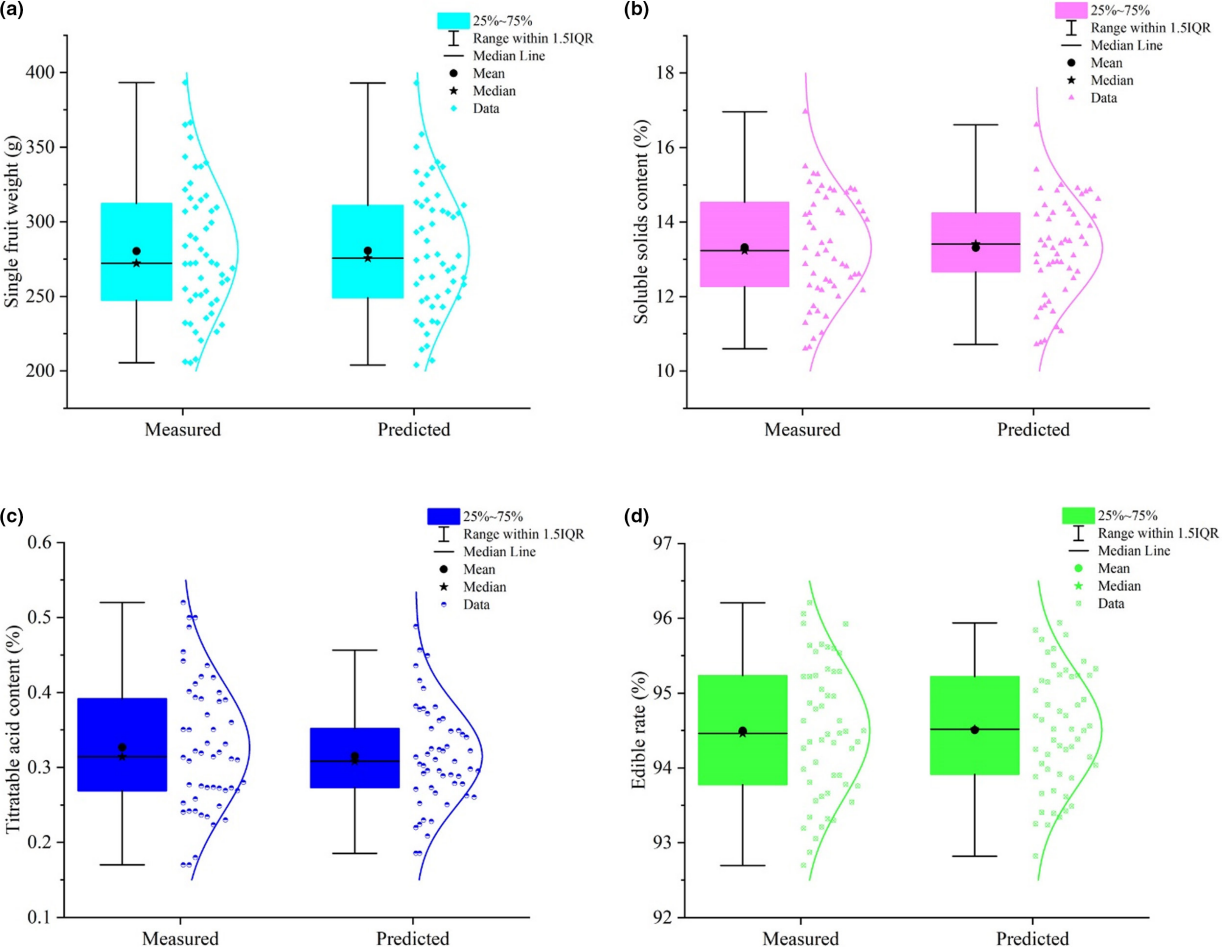
The ANN model of fruit quality. (a) Box plot and scatter plot with normal curve of predicted and measured single fruit weight values; (b) Box plot and scatter plot with normal curve of predicted and measured soluble solid content values; (c) Box plot and scatter plot with normal curve of predicted and measured titratable acid content values; (d) Box plot and scatter plot with normal curve of predicted and measured fruit edible rate values

To further explore the relationship between the content of mineral elements in soil and fruit quality, we established a model using ANNs and predicted fruit quality through the content of mineral elements in soil. To develop a reliable prediction model of single fruit weight, we used five different training functions and three different transfer functions to evaluate the prediction performance of the ANN models (Table 3). Meanwhile, we also tested the structure of the hidden layer in the neural network model to obtain an accurate prediction model. The models established by different training functions have great differences. When the Log‐Sigmoid transfer function was used, the prediction accuracy of the LM training function was 0.9735 at the highest, which was significantly higher than the predicted value of the other four training functions. When the Liner transfer function was used, the maximum prediction accuracy of the SCG training function was 0.6093, which was not different from that of the other four training functions. When the Tangent‐sigmoid transfer function was used, the maximum prediction accuracy of the CGB training function was 0.7488. In addition, the models constructed by different transfer functions differ greatly. Except for the LM training function, the prediction accuracy of the other four training functions was higher than that of the other two transfer functions when they use the Tangent‐sigmoid transfer function to establish the model. The ANN model with Log‐sigmoid transfer function and LM training function shows the best prediction performance in the comprehensive evaluation. The prediction accuracy *R* ^2^ was the highest (.9735), and the model evaluation coefficients RMSE (0.0482), *MSE* (0.0023), MAE (0.0308), RSE (0.1155), and MAPE (0.0631) were the lowest. Meanwhile, in order to more intuitive to show prediction performance of this model, we also compared the single fruit weight of the predicted value and measured values (Figure 2a), the results showed that their distribution patterns were very close, and their box graph structure was almost the same, indicated that ANN model could accurately predict the single fruit weight of peach.

**TABLE 3:** Different ANN models for predicting the peach single fruit weight

Training function	Transfer function	Best model	*R* ^2^	RMSE	*MSE*	MAE	RSE	MAPE
BFG	Log‐sigmoid	10–9–1	.5184	0.1907	0.0364	0.1525	0.4567	0.3342
	Linear	10–10–1	.5737	0.2055	0.0422	0.1626	0.4923	0.3376
	Tangent‐sigmoid	10–10–1	.6111	0.1907	0.0364	0.1493	0.4568	0.3231
CGB	Log‐sigmoid	10–10–1	.6289	0.1918	0.0368	0.1510	0.4594	0.3210
	Linear	10–10–1	.5223	0.1955	0.0382	0.1571	0.4682	0.3425
	Tangent‐sigmoid	10–9–1	.7488	0.1869	0.0349	0.1509	0.4477	0.3274
CGP	Log‐sigmoid	10–9–1	.6072	0.1863	0.0347	0.1446	0.4461	0.3013
	Linear	10–11–1	.5587	0.1939	0.0376	0.1532	0.4645	0.3339
	Tangent‐sigmoid	10–10–1	.6275	0.1902	0.0362	0.1514	0.4557	0.3240
LM	Log‐sigmoid	10–11–1	.9735	0.0482	0.0023	0.0308	0.1155	0.0631
	Linear	10–12–1	.5993	0.1940	0.0377	0.1516	0.4648	0.3245
	Tangent‐sigmoid	10–11–1	.6656	0.1785	0.0319	0.1459	0.4276	0.2957
SCG	Log‐sigmoid	10–11–1	.5751	0.1951	0.0381	0.1521	0.4672	0.3185
	Linear	10–9–1	.6093	0.1911	0.0365	0.1507	0.4576	0.3297
	Tangent‐sigmoid	10–8–1	.6231	0.1966	0.0386	0.1638	0.4708	0.3691

Note

The abbreviations are same below for Tables 4, 5, 6.

Abbreviations: BFG, BFGS Quasi‐Newton; CGB, Conjugate Gradient with Powell/Beale Restarts; CGP, Polak–Ribiére Conjugate Gradient; LM, Levenberg–Marquardt; SCG, Scaled Conjugate Gradient.

### 3.3 ANNs model for predicting the soluble solid content

Similarly, we established a prediction model to accurately predict the soluble solid content of fruit based on the content of soil mineral elements (Table 4). When the Log‐sigmoid transfer function was used, the prediction accuracy of LM transfer function was the highest (0.9607), which was significantly higher than that of the other four training functions. When the Liner transfer function was used, the five training functions have lower predictive accuracy than those of the other two transfer functions. When the Tangent‐sigmoid transfer function was used, the LM transfer function had the highest predictive accuracy (0.8874). The prediction accuracy *R* ^2^ of ANN model with log‐sigmoid transfer function and Levenberg–Marquardt training function was the highest (.9607), and the model evaluation coefficients RMSE (0.0598), *MSE* (0.0036), MAE (0.0401), RSE (0.1432), and MAPE (0.1501) were the lowest. Meanwhile, we also compared the predicted and measured values of soluble solid content (Figure 2b), and the results showed that their distribution patterns were very similar, and they had similar box diagram structures, indicating that the established ANN model could accurately predict the soluble solid content of fruits.

**TABLE 4:** Different ANN models for predicting the peach soluble solid content

Training function	Transfer function	Best model	*R* ^2^	RMSE	*MSE*	MAE	RSE	MAPE
BFG	Log‐sigmoid	10–9–1	.8201	0.1423	0.0202	0.1205	0.3408	0.8117
	Linear	10–9–1	.7348	0.1545	0.0239	0.1222	0.3700	1.3263
	Tangent‐sigmoid	10–10–1	.8240	0.1276	0.0163	0.1009	0.3056	0.8077
CGB	Log‐sigmoid	10–11–1	.7941	0.1450	0.0210	0.1150	0.3473	1.0120
	Linear	10–11–1	.7535	0.1511	0.0228	0.1212	0.3620	1.1116
	Tangent‐sigmoid	10–10–1	.7647	0.1391	0.0193	0.1135	0.3331	1.0256
CGP	Log‐sigmoid	10–12–1	.7951	0.1391	0.0193	0.1192	0.3331	1.2317
	Linear	10–9–1	.7452	0.1489	0.0222	0.1176	0.3567	1.0473
	Tangent‐sigmoid	10–10–1	.7719	0.1427	0.0204	0.1173	0.3418	0.4902
LM	Log‐sigmoid	10–11–1	.9607	0.0598	0.0036	0.0401	0.1432	0.1501
	Linear	10–9–1	.7400	0.1481	0.0219	0.1188	0.3548	1.1084
	Tangent‐sigmoid	10–11–1	.8874	0.1124	0.0126	0.0870	0.2692	0.7542
SCG	Log‐sigmoid	10–10–1	.7968	0.1430	0.0205	0.1175	0.3426	0.9364
	Linear	10–12–1	.7190	0.1502	0.0226	0.1219	0.3598	0.9670
	Tangent‐sigmoid	10–10–1	.8189	0.1377	0.0190	0.1133	0.3299	1.2719

### 3.4 ANNs model for predicting the titratable acid content

Similarly, using the content of soil mineral elements, we established a prediction model to accurately predict the fruit titratable acid content (Table 5). When the Log‐sigmoid transfer function was used, the prediction accuracy of LM transfer function was the highest (0.9036), which was significantly higher than that of other four training functions. When the Liner transfer function was used, the five training functions have lower predictive accuracy than those of the other two transfer functions. When the Tangent‐sigmoid transfer function was used, the LM transfer function had the highest predictive accuracy (0.8796). The prediction accuracy *R* ^2^ of ANN model with log‐sigmoid transfer function and Levenberg–Marquardt training function was the highest (.9036), and the model evaluation coefficients RMSE (0.1045), *MSE* (0.0109), MAE (0.0765), RSE (0.2502), and MAPE (0.2056) were the lowest. Their distribution patterns of predicted and measured values (Figure 2c) were very similar, and they had similar box diagram structures, indicating that the established ANN model could accurately predict the titratable acid content of fruits.

**TABLE 5:** Different ANN models for predicting the peach titratable acid content

Training function	Transfer function	Best model	*R* ^2^	RMSE	*MSE*	MAE	RSE	MAPE
BFG	Log‐sigmoid	10–9–1	.7720	0.1927	0.0371	0.1554	0.4616	0.3699
	Linear	10–11–1	.7130	0.1936	0.0375	0.1603	0.4637	0.4652
	Tangent‐sigmoid	10–12–1	.7880	0.1799	0.0324	0.1391	0.4309	0.3568
CGB	Log‐sigmoid	10–11–1	.7599	0.1774	0.0315	0.1487	0.4250	0.4217
	Linear	10–8–1	.7539	0.1927	0.0371	0.1533	0.4615	0.4198
	Tangent‐sigmoid	10–12–1	.8204	0.1934	0.0374	0.1487	0.4631	0.3944
CGP	Log‐sigmoid	10–10–1	.7287	0.1944	0.0378	0.1536	0.4657	0.4048
	Linear	10–8–1	.7530	0.1947	0.0379	0.1569	0.4664	0.4283
	Tangent‐sigmoid	10–8–1	.8432	0.1906	0.0363	0.1475	0.4565	0.3720
LM	Log‐sigmoid	10–11–1	.9036	0.1045	0.0109	0.0765	0.2502	0.2056
	Linear	10–9–1	.7724	0.1947	0.0379	0.1529	0.4663	0.4251
	Tangent‐sigmoid	10–9–1	.8796	0.1566	0.0245	0.1166	0.3752	0.3949
SCG	Log‐sigmoid	10–9–1	.8179	0.1753	0.0307	0.1441	0.4198	0.4186
	Linear	10–11–1	.7535	0.1942	0.0377	0.1577	0.4652	0.4544
	Tangent‐sigmoid	10–8–1	.8365	0.1780	0.0317	0.1432	0.4262	0.3521

### 3.5 ANN model for predicting the fruit edible rate

Similarly, we established a prediction model to accurately predict the fruit edible rate by the content of soil mineral elements (Table 6). When using the Log‐sigmoid transfer function, the LM transfer function had the highest prediction accuracy (0.9440), which was significantly higher than that of the other four training functions. When the Liner transfer function was used, the five training functions have lower predictive accuracy than those of the other two transfer functions. When the Tangent‐sigmoid transfer function was used, the LM transfer function had the highest predictive accuracy (0.8299). The prediction accuracy *R* ^2^ of ANN model with log‐sigmoid transfer function and Levenberg–Marquardt training function was the highest (.9440), and the model evaluation coefficients RMSE (0.0917), *MSE* (0.0084), MAE (0.0688), RSE (0.2195), and MAPE (0.1998) were the lowest. Meanwhile, the comparison between the predicted and measured values of titratable acid content (Figure 2d) shows that their distribution patterns are very similar, and their box diagram structures were the same, indicating that the established ANN model could accurately predict the edible rate of fruits.

**TABLE 6:** Different ANN models for predicting the peach edible rate

Training function	Transfer function	Best model	*R* ^2^	RMSE	*MSE*	MAE	RSE	MAPE
BFG	Log‐sigmoid	10–10–1	.7674	0.2144	0.0460	0.1677	0.5136	0.5263
	Linear	10–11–1	.7241	0.2274	0.0517	0.1864	0.5448	0.5961
	Tangent‐sigmoid	10–8–1	.7631	0.2280	0.0520	0.1776	0.5460	0.5281
CGB	Log‐sigmoid	10–12–1	.6954	0.2253	0.0508	0.1812	0.5397	0.6082
	Linear	10–11–1	.6603	0.2289	0.0524	0.1915	0.5483	0.5752
	Tangent‐sigmoid	10–10–1	.7622	0.2118	0.0449	0.1683	0.5073	0.5499
CGP	Log‐sigmoid	10–12–1	.7111	0.2336	0.0546	0.1980	0.5594	0.5657
	Linear	10–12–1	.7283	0.2203	0.0485	0.1792	0.5276	0.5563
	Tangent‐sigmoid	10–10–1	.7512	0.2439	0.0595	0.1966	0.5841	0.5853
LM	Log‐sigmoid	10–9–1	.9440	0.0917	0.0084	0.0688	0.2195	0.1998
	Linear	10–12–1	.7138	0.2289	0.0524	0.1828	0.5482	0.5941
	Tangent‐sigmoid	10–11–1	.8299	0.2007	0.0403	0.1227	0.4807	0.3806
SCG	Log‐sigmoid	10–9–1	.7389	0.2156	0.0465	0.1774	0.5163	0.5614
	Linear	10–9–1	.7153	0.2267	0.0514	0.1811	0.5429	0.5239
	Tangent‐sigmoid	10–9–1	.6976	0.2237	0.0501	0.1839	0.5359	0.5785

### 3.6 The sensitivity analysis of the soil mineral elements on the peach fruit quality

We obtained reliable prediction models by constructing the ANN model, which can accurately predict the fruit quality indexes based on the content of mineral elements in the soil. To elucidate the influence of certain soil elements on fruit quality indicators, we conducted a sensitivity analysis of the prediction model to explore which mineral elements had a greater impact on fruit quality indicators. In the sensitivity analysis, RMSE value represented the relative contribution of the input value to the output value in the ANN model. The higher the RMSE value was, the higher the importance of the eliminated input value was, that is, the greater the influence of the eliminated orchard soil mineral elements on the fruit quality. In the prediction model of single fruit weight, RMSE values from large to small were B, Ca, N, K, P, Fe, Mn, Mg, Zn, and Cu, indicating that available B, Ca, N, and K content in the soil had the greatest influence on the single fruit weight. In the prediction model of fruit soluble solid content, RMSE values from the largest to the smallest were Fe, K, B, Ca, Mn, P, N, Mg, Cu, and Zn, indicating that available Fe, K, B, and Ca content in the soil had the greatest effect on fruit soluble solid content. In the prediction model of fruit titratable acid content, RMSE values from large to small were Ca, N, B, K, P, Fe, Mg, Zn, Mn, and Cu, indicating that available Ca, N, B, and K content in the soil had the greatest influence on the fruit titratable acid content. In the prediction model of fruit edible rate, RMSE values from the largest to the smallest were Ca, Fe, N, Mn, P, K, Mg, B, Cu, and Zn, indicating that available Ca, Fe, N, and Mn content in soil had the greatest effect on fruit edible rate. Finally, the content of available N, K, Ca, Fe, and B in the soil greatly influences fruit quality indexes Table 7.

**TABLE 7:** The sensitivity analysis of the peach orchard soil mineral elements on fruit quality

ANN model	Single fruit weight		Soluble solid content		Titratable acid content		Edible rate	
	*R* ^2^	RMSE	*R* ^2^	RMSE	*R* ^2^	RMSE	*R* ^2^	RMSE
ANN without N	.6737	0.4387	.7638	0.1637	.7126	0.4169	.7624	0.5227
ANN without P	.7872	0.4356	.8036	0.2236	.6889	0.3452	.7556	0.4780
ANN without K	.6483	0.4360	.8201	0.2959	.7240	0.3608	.7272	0.3881
ANN without Ca	.7015	0.4433	.7380	0.2550	.6147	0.4457	.7264	0.6368
ANN without Mg	.7126	0.2228	.9084	0.1585	.8070	0.2408	.7295	0.3534
ANN without Fe	.6947	0.4336	.7124	0.3134	.7094	0.2890	.7443	0.6244
ANN without Mn	.7159	0.4157	.8104	0.2246	.8286	0.1966	.7563	0.4790
ANN without Cu	.8141	0.1621	.8552	0.1260	.8848	0.1156	.8028	0.2790
ANN without Zn	.7647	0.1970	.8211	0.1189	.8024	0.2317	.8008	0.1944
ANN without B	.7349	0.5624	.6919	0.2636	.7139	0.3916	.7478	0.2813

### 3.7 Response surface methodology analysis

**FIGURE 3:**
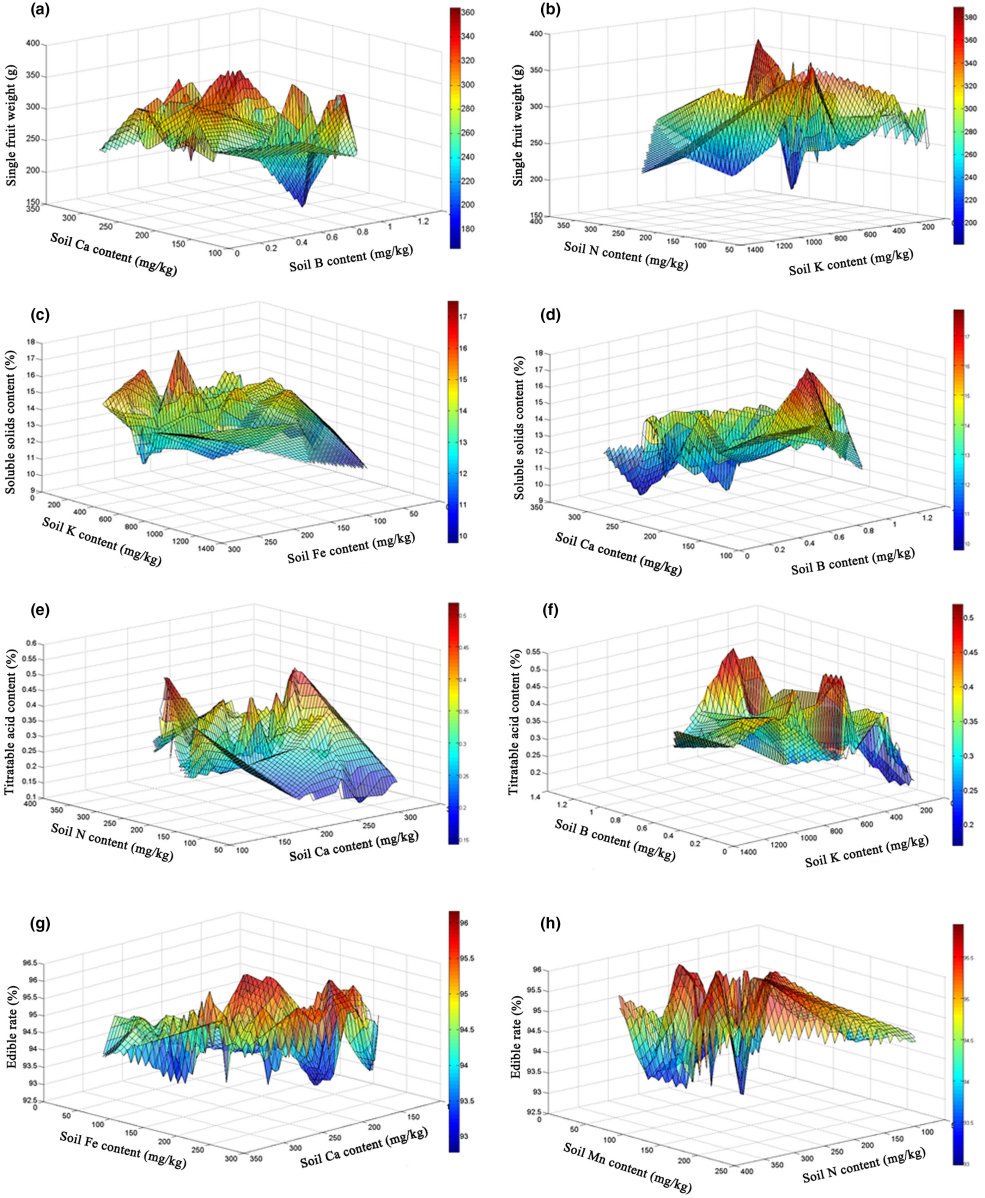
Response surface methodology of fruit quality and orchard soil mineral element content. (a) Soil available B, Ca content, and single fruit weight; (b) Soil available N, K content, and single fruit weight; (c) Soil available Fe, K content, and soluble solid content; (d) Soil available B, Ca content, and soluble solid content; (e) Soil available Ca, N content, and titratable acid content; (f) Soil available B, K content, and titratable acid content; (g) Soil available Ca, Fe content, and edible rate; (h) Soil available N, Mn content, and edible rate

According to the above ANN models' sensitivity analysis results, the content of some mineral elements in the soil had significant effect on fruit quality. To further explore the suitable range of these elements, we carried out a response surface analysis. The response surface analysis of soil available B, Ca content, and single fruit weight is shown in Figure 3a. When soil available B content was 0.2–0.96 mg/kg, available Ca content was 204.0–296.0 mg/kg, a higher single fruit weight can be obtained. When soil available B content was 0.8–1.2 mg/kg, available Ca content was 130.0–156.0 mg/kg, a higher single fruit weight can also be obtained. The response surface analysis of soil available N, K content, and single fruit weight in the soil is shown in Figure 3b. When available N content in the soil was 116.0–182.0 mg/kg, available K content was 450.0–600.0 mg/kg, a high single fruit weight can be obtained. When available N content in the soil was 204.0–276.0 mg/kg, available K content was 490.0–585.0 mg/kg, a higher single fruit weight can be obtained. However, when available K content in the soil was greater than 910.0 mg/kg, the single fruit weight index decreased significantly. The response surface analysis of available Fe, K content in the soil, and soluble solid content is shown in Figure 3c. When soil available Fe content was 196.0–272.0 mg/kg and available K content was 218.0–391.0 mg/kg, higher soluble solid content can be obtained. When soil available Fe content was 60.0–140.0 mg/kg and available K content was 400.0–836.0 mg/kg, higher soluble solid content can also be obtained. However, when the content of available Fe in the soil was lower than 50.0 mg/kg, the content of soluble solids decreased significantly. The response surface analysis of available B, Ca content in the soil, and soluble solid content is shown in Figure 3d. When available B content in the soil was 0.58–0.90 mg/kg and available Ca content was 140.0–174.0 mg/kg, higher soluble solid content can be obtained. However, when available Ca content in the soil was greater than 266.0 mg/kg, soluble solid content decreases significantly. The response surface analysis of available Ca, N in the soil, and titratable acid content is shown in Figure 3e. When available Ca content in the soil was 168.0–306.0 mg/kg, available N content was 62.0–108.0 mg/kg, lower titratable acid content can be obtained. When available Ca content in the soil was 168.0–198.0 mg/kg, and available N content was 71.0–176.0 mg/kg, lower titratable acid content can be obtained. The response surface analysis of available B, K in the soil, and titratable acid content is shown in Figure 3f. When the content of available B in the soil was 0.16–0.62 mg/kg, available K content was 80.0–160.0 mg/kg, the lower titratable acid content can be obtained. When available B content in the soil was 0.78–1.06 mg/kg, available K content was 240.0–360.0 mg/kg, the lower titratable acid content can be obtained. While available B content in the soil was 0.80–1.10 mg/kg, available K content was 720.0–1000.0 mg/kg, the lower titratable acid content can also be obtained. The response surface analysis of available Ca, Fe content in the soil, and edible rate is shown in Figure 3g. When available Ca content in the soil was 174.0–234.0 mg/kg, available Fe content was 220.0–270.0 mg/kg, higher edible rate can be obtained. When available Ca content in the soil was 170.0–240.0 mg/kg, available Fe content was 130.0–170.0 mg/kg, higher edible rate can also be obtained. The response surface analysis of available N, Mn content in the soil, and single fruit weight is shown in Figure 3h. When available N content in the soil was 140.0–210.0 mg/kg, available Mn content was 105.0–152.0 mg/kg, higher edible rate can be obtained. When available N content in the soil was 220.0–250.0 mg/kg, available Mn content was 37.0–72.0 mg/kg, higher edible rate can also be obtained.

In conclusion, peach fruit quality indexes can be significantly improved when the content of available N was 71–108 mg/kg, available K was 490.0–585.0 mg/kg, available Ca was 170.0–198.0 mg/kg, available Fe content was 125–140 mg/kg, and available B was 0.80–1.02 mg/kg in the soil.

## 4 DISCUSSION

### 4.1 The ANN models building and interpretation

The relationship between fruit quality indexes and orchard soil mineral elements was complex and cannot be accurately revealed using conventional modeling techniques or mathematical methods. In recent years, an increasing number of researchers have used the ANN model as a forecasting tool for a variety of subjects, including agricultural research (Abdipour et al., 2019; Huang et al., 2021; Mazen et al., 2019; Torkashvand et al., 2019; Zhang et al., 2020), indicating that the ANN model was a highly effective forecasting tool. In essence, the neural network realized a mapping function from input to output. The mathematical theory proved that the three‐layer neural network could approximate any nonlinear continuous function with any precision. This made it particularly for solving complex internal mechanisms, and had strong nonlinear mapping ability. During the training stage, the ANN can extract the input and output data automatically, and memorize the learning content in the network's weights according to reasonable rules, showing a high degree of self‐learning and adaptive ability. The neural network had a certain fault‐tolerant ability, which will not affect the global training results when some of its neurons were destroyed (Alvarez et al., 2009; Kumar et al., 2009).

In this study, we built the ANN models by using different training functions and transfer functions, and constantly tested the hidden layer structure. Finally, we obtained four reliable prediction models which can accurately predict fruit quality index of peach. Among them, the topological structure of single fruit weight prediction model was 10–11–1, that of soluble solid content was 10–11–1, that of titratable acid content was 10–11–1, and that of edible rate was 10–9–1, with the highest *R* ^2^ value of .9735, .9607, .9036, and .9440, other errors were also the lowest. We also found that the best prediction models used Levenberg–Marquardt training function and Log‐Sigmoid transfer function. Many researchers used sigmoid transfer function to predict the relevant indicators of different crops. Belouz et al. (2022) showed that An ANN model with a 12–34–1 topology could more accurately predict tomato yield. Ray et al. (2020) showed that the ANN model with 18–5–1 structure is the best model for predicting the coronarin D content. In addition, we also compared the measured values with the predicted values of the ANN models by scatter plot and box plot, the distribution patterns of the two were almost the same, which further verified the reliability and accuracy of the constructed models.

### 4.2 The importance of soil mineral nutrients to fruit quality

Fruit quality was one of the market's most important core competitiveness factor, which not only affected fruit price, but also fruit sales volume (Cun et al., 2020). It was caused by a combination of multiple factors, especially the individual and combined effects of mineral nutrients (Aular et al., 2013). The soil was a vital component in the ecosystem's exchange of matter and energy. The abundance and deficiency of soil nutrients had a significant effect on fruit tree growth and development as well as fruit yield and quality (Gao, 2015). Abundant soil mineral nutrients can promote the healthy growth of fruit trees and play a crucial role in fruit quality (Jin et al., 2010).

In the present study, soil available B content had the greatest effect on fruit weight. B could promote carbohydrate transformation and translocation and accelerate plant growth and development. When B was in abundant supply, the plant was thriving, the root system was good, and the harvest was assured. Otherwise, it can lead to poor plant growth, reduced product quality and yield. Moreover, it was also a participant in sugar transport and metabolism, which had impacted on fruit quality (Fan et al., 2016; Wu, 2020). Additionally, the amount of available Ca content also has a significant impact on single fruit weight. Ca was an important component of plant cell wall, which can promote the division of epidermal cells, improved the toughness and thickness of fruit epidermis, thus accelerating the growth of fruit epidermis and promoting fruit development (Zocchi, 1995). As a result, the content of available Ca was also the most important factor affecting fruit edible rate. When Ca was deficient, it can inhibit the ability of the root to absorb nutrients, lead to plant growth decline, prone to premature senescence, and affect the photosynthesis of plants (Xin, 2008). The results of response surface analysis also showed that when the Ca content was lower than 168 mg/kg, the titratable acid content of fruit would increase. Soil available Fe and K content had the greatest effect on soluble solid content. For plants, Fe was one of the elements of chlorophyll, which participates in photosynthesis and produces organic matter such as carbohydrates (Jia, 2019). Fe fertilizer treatment can promote the accumulation of soluble sugar and soluble solid content in fruit, which was conducive to the improvement of fruit quality (Guo et al., 2017). K was the activator of more than 60 enzymes, such as synthetase, dehydrogenase, and transporter. It participated in the synthesis and transportation of protein, starch, sugar, and other substances (Pettigrew, 2008). For many horticultural plants, adequate K supply can increase fruit volume, yield, soluble solid content, VC content, and other nutrients (Quaggio et al., 2011; Szewczuk et al., 2011). On the one hand, it promoted protein synthesis and increased the sugar content of fruit by transporting photosynthate from leaf to fruit. On the other hand, it also aided in the conversion of starch to sugar in the fruit, increasing the sugar content of fruit and accelerating fruit ripening (Wu, 2016). As for the relative contribution, available N, K, Ca, Fe, and B contents in the soil greatly influence fruit quality indexes of peach.

## 5 CONCLUSION

The ANN methods were used in this study to establish prediction models to explore the effect of soil mineral element content on peach fruit quality. The results indicated that when the prediction model structure of the single fruit weight, the soluble solid content, and the titratable acid content was 10–11–1, and the edible rate prediction model was 10–9–1, which can achieve the highest accuracy (*R* ^2^ = .9735, .9607, .9036, and .9440, respectively). The sensitivity analysis results showed that soil available N, K, Ca, Fe, and B content contributed the most to the quality of peach fruit. The response surface methodology analysis confirmed the suitable range of these mineral elements, when the content of available N was 71–108 mg/kg, available K was 490.0–585.0 mg/kg, available Ca was 170.0–198.0 mg/kg, available Fe content was 125–140 mg/kg, and available B content was 0.80–1.02 mg/kg in the soil, peach fruit quality indexes can be significantly improved.

## CONFLICTS OF INTEREST

The authors declare that they have no conflict of interest.

## ETHICS APPROVAL

This article does not contain any studies with animal or human subject.

## Data Availability

The original contributions presented in the study are included in the article, and further inquiries can be directed to the corresponding authors.
